# SG-APSIC1200: Infection prevention control along the fast track: Supporting the construction, operation, and closure of a COVID-19 community treatment facility in an F1 Pit

**DOI:** 10.1017/ash.2023.28

**Published:** 2023-03-16

**Authors:** Wee Ling Tee, Somani Jyoti, Razali Bin Mahdi, Cathrine Teo

**Affiliations:** National University Hospital, Singapore; Singapore National University Hospital, Singapore; Singapore National University Hospital, Singapore; Singapore National University Hospital, Singapore

## Abstract

**Objectives:** The infection prevention team (IPT) was tasked with providing technical guidance for the construction and setup of a community treatment facility in 3 weeks at a Formula 1 (F1) racing pit to house elderly SARS-CoV-2–positive cases. **Methods:** The facility was setup with 737 beds including an isolation unit and a resuscitation bay. The multidisciplinary team decided on zone segregation (ie, green and hot zones) and discussed the clean–dirty workflow. IPC measures were revisited, especially regarding the layout of the donning and doffing station, as the facility expanded to accommodate patients with more comorbidities and those who needed dialysis. IPC training for nominated infection control liaison officers (ICLOs) was conducted using a “train the trainer” approach for mask fitting, hand hygiene, donning and doffing of personal protective equipment (PPE). Enhanced IPC measures, including weekly audit and staff surveillance, were mandatory, and monitoring was performed according to MOH guidelines. Linen and waste management and the cleaning and disinfection process were established at the beginning of the project. **Results:** Construction was completed within 3 weeks. The setup was completed in November 2021 for 737 beds. There were 758 admissions during the 4-month operation. In total, 12 trained ICLOs oversaw the training of 200 healthcare workers. They conducted 12 IPC audits and provided feedback to all staff. Compliance with PPE practices was inconsistent, and findings were shared during daily after-action reviews for improvement. The greatest challenges were converting the F1 facility to a healthcare facility, training staff with no IPC knowledge, and monitoring IPC on the ground. The trained ICLOs were successful in implementing, practicing, and monitoring IPC measures with minimal assistance from the infection prevention team. **Conclusions:** Operation began on November 5, 2021, and ceased on March 9, 2022. The community treatment facility construction, setup, and operations were completed within a short timeframe due to the efforts of various stakeholders. We faced many challenges, but we managed to implement and uphold IPC standards from beginning to end.

